# Site-specific variation in gene expression from *Symbiodinium* spp. associated with offshore and inshore *Porites astreoides* in the lower Florida Keys is lost with bleaching and disease stress

**DOI:** 10.1371/journal.pone.0173350

**Published:** 2017-03-29

**Authors:** Briana Hauff Salas, Joshua A. Haslun, Kevin B. Strychar, Peggy H. Ostrom, James M. Cervino

**Affiliations:** 1 University of Texas School of Medicine, San Antonio, TX, United States of America; 2 Michigan State University, Department of Integrative Biology, East Lansing, MI, United States of America; 3 Annis Water Resources Institute-Grand Valley State University, Muskegon, MI, United States of America; 4 Woods Hole Oceanographic Institute, Department of Marine Chemistry & Geochemistry, Woods Hole, MA, United States of America; University of Bologna, ITALY

## Abstract

Scleractinian coral are experiencing unprecedented rates of mortality due to increases in sea surface temperatures in response to global climate change. Some coral species however, survive high temperature events due to a reduced susceptibility to bleaching. We investigated the relationship between bleaching susceptibility and expression of five metabolically related genes of *Symbiodinium* spp. from the coral *Porites astreoides* originating from an inshore and offshore reef in the Florida Keys. The acclimatization potential of *Symbiodinium* spp. to changing temperature regimes was also measured *via* a two-year reciprocal transplant between the sites. Offshore coral fragments displayed significantly higher expression in *Symbiodinium* spp. genes *PCNA*, *SCP2*, *G3PDH*, *PCP* and *psaE* than their inshore counterparts (p<0.05), a pattern consistent with increased bleaching susceptibility in offshore corals. Additionally, gene expression patterns in *Symbiodinium* spp. from site of origin were conserved throughout the two-year reciprocal transplant, indicating acclimatization did not occur within this multi-season time frame. Further, laboratory experiments were used to investigate the influence of acute high temperature (32°C for eight hours) and disease (lipopolysaccharide of *Serratia marcescens*) on the five metabolically related symbiont genes from the same offshore and inshore *P*. *astreoides* fragments. Gene expression did not differ between reef fragments, or as a consequence of acute exposure to heat or heat and disease, contrasting to results found in the field. Gene expression reported here indicates functional variation in populations of *Symbiodinium* spp. associated with *P*. *astreoides* in the Florida Keys, and is likely a result of localized adaptation. However, gene expression patterns observed in the lab imply that functional variation in zooxanthellae observed under conditions of chronic moderate stress is lost under the acute extreme conditions studied here.

## Introduction

A decline in global coral populations as high as 50% has been recorded over the last 30–40 years, with strong links to coral bleaching [1–3]. Bleaching is a response to elevated seawater temperatures and high irradiance, and results when the density of symbiotic algae (*Symbiodinium* spp. commonly referred to as zooxanthellae) declines severely in the host coral allowing the white skeleton below to show [4,5]. Considering that coral hosts derive up to 95% of their daily metabolic needs from zooxanthellae [6], the loss of symbionts compromises the host during a bleaching event. Depending on the extent and duration of a bleaching event, loss of zooxanthellae may lead to coral host mortality [7]. Given the importance of the host-zooxanthellae symbiosis, plasticity in algal response to stress is an important area of coral research [8–11].

Coral susceptibility to bleaching is influenced by the *Symbiodinium* spp. clade type harbored by the host [12]. For example, Sampayo *et al*. [13] demonstrated that bleaching susceptibility of *Stylophora pistillata* colonies was determined by compositional differences at the subclade level within *Symbiodinium* spp. of clade C. In the Florida Keys, several studies found variation in bleaching susceptibility between inshore and offshore communities of *P*. *astreoides* [14–16]. The observation of variation in subclades between proximate inshore and offshore reefs prompts questions of the role of genetically associated functional variation in zooxanthellae to bleaching susceptibility [17]. However, the role of zooxanthellae in bleaching susceptibility variation in inshore and offshore *P*. *astreoides* of the Florida Keys is relatively unknown [15].

Coral diseases are a second leading stressor threatening the mortality of coral reefs [18], and can be exacerbated when combined with bleaching stress [19,20]. As with bleaching, recent studies demonstrate variation in zooxanthellae susceptibility to disease stress, [13,17,20] and therefore may play an important role in coral survival to both bleaching and disease. However, these studies assessed stress responses of zooxanthellae in culture, or under conditions of chronic moderate stress, but not *in vivo* under acute, extreme stress most commonly associated with mass mortality events. Here, chronic moderate stress refers to elevated levels of a stressor experienced over a lengthy period of time (i.e. weeks or months). Chronic moderate stress will produce a response such as bleaching, but may not result in death [21]. Acute, extreme stress is often associated with short periods of drastically elevated temperatures, but may also be related to extremes in irradiance, disease, or the synergistic effect of two or more environmental variables [22]. Herein, we differentiate chronic and acute stress on the basis of time.

Scleractinian coral have recently experienced up to 80% mortality in the Florida Keys as a result of the separate and combined effects of bleaching and disease [23]. Acroporid serratosis refers to White Pox Disease specifically caused by *Serratia marcescens* [24]. *S*. *marcescens* is derived from the gut of animals, including humans, and was identified in wastewater of the Florida Keys [25]. Acroporid serratosis has caused a 70% loss of *Acropora palmata* species in the Florida Keys [24]. The continued success of coral reefs in the Florida Keys is therefore likely dependent on the ability of zooxanthellae to survive acute extreme stress and the synergistic effects of bleaching and disease [3,20].

Changes in metabolic processes as markers of bleaching susceptibility have a long-standing history in the study of zooxanthellae response to stress [11,26–29]. Changes in metabolism are commonly assessed *via* gene expression analysis, which targets genes related to the production of metabolically important proteins. Common examples include, proliferating cell nuclear antigen (*PCNA*), a protein involved in DNA synthesis and replication [30], non-specific lipid-transfer protein (*SCP2*), which mediates the transfer of all common lipids [31], Glyceraldehyde-3-phosphate dehydrogenase (*G3PDH*), an enzyme involved in glycolysis [32], peridinin chlorophyll alpha binding protein I chloroplastic (*PCP*) and photosystem I reaction center subunit IV (*psaE*). *PCP* is involved in the capture of solar energy [33] and *psaE* stabilizes interactions within the photosystem 1 complex [34]. Variation in the expression of these metabolic genes affords functional variability to zooxanthellae and can be involved in acclimatization to bleaching and disease conditions [35–37].

Here, we studied the role of zooxanthellae in the bleaching susceptibility of *P*. *astreoides* by evaluating symbiont gene expression in reciprocal transplants of *P*. *astreoides* in the Florida Keys. The expression of zooxanthellae genes *PCNA*, *SCP2*, *G3PDH*, *PCP* and *psaE* was measured in coral sub-samples from inshore and offshore reefs differing in thermal and irradiance regimes [16,17]. Differences in patterns of gene expression in the reciprocal transplant allowed us to evaluate the role of these genes in acclimatization to environmental stress. Expression of the same symbiont genes was measured in response to a laboratory study combining bleaching temperatures and lipopolysaccharide (LPS) from *S*. *marcescens* in order to determine the synergistic effects of acute (8-hour) heat and LPS exposure. Lipopolysaccharides are large molecules found on the outer membrane of gram-negative bacteria [38]. When present, LPS elicits a strong immune response in infected organisms [39]. Variation in the response of zooxanthellae to acute stress in the laboratory compared to the chronic moderate stress experienced in the field allowed inferences of the role of the coral host in response to acute stress. To the author’s knowledge, the effects of LPS on the expression of zooxanthellae genes have not been described previously.

## Materials and methods

### Reciprocal transplant and sample collection

In this study, *Symbiodinium* spp. from *P*. *astreoides* reciprocally transplanted from an inshore and offshore site in the lower Florida Keys were analyzed for changes in gene expression over two years. Study sites, coral collection, reciprocal transplant design and sample collection for analyses were completed as outlined in Hauff *et al*. [17]. Briefly, in September 2011, ten 16x16 cm fragments of *P*. *astreoides* were collected from Birthday reef (inshore: 24.57917N -81.49693W) and an additional ten 16x16 cm fragments were obtained from Acer24 reef (offshore: 24.55268N -81.43741W)(Permit #FKNMS-2011-107, Florida Keys National Marine Sanctuary). Birthday reef and Acer24 reef are patch reefs separated by Hawk Channel and have notably distinct temperature and turbidity regimes, but are otherwise similar (i.e. depth, species diversity). Relative to Acer24 reef, Birthday reef has higher turbidity, lower light, higher annual average temperatures (~1°C) and lower bleaching (for a detailed description of site specific parameters see Hauff *et al*. 2016, Haslun *et al*., 2016).

**Fig 1:**
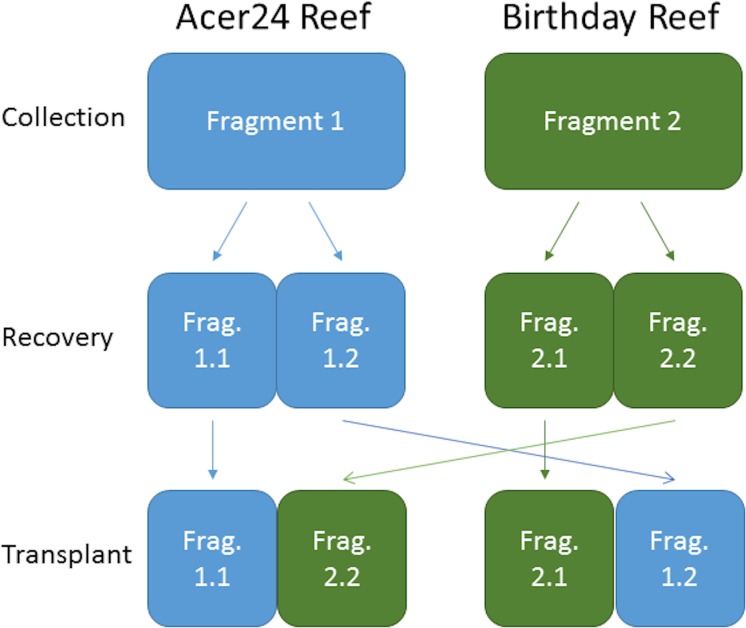
Graphical representation of reciprocal transplant sampling design. A 16x16 cm fragment of *P*. *astreoides* was collected from each reef. This fragment was sectioned into two 16x8 cm small fragments and allowed to recover. Coral was then placed back onto a reef with one small fragment being placed at its reef of origin, while the other fragment half was transplanted to the companion site. For the above, fragments colored in blue originated from Acer24 Reef, while the fragments colored in green originated from Birthday Reef. The above was repeated ten times for each reef, for a total of n = 20 *P*. *astreoides* sampled and n = 40 small fragments placed back on to reefs.

The 20 *P*. *astreoides* fragments taken from Birthday reef and Acer24 reef were sectioned into nearly equal halves. These small fragments were approximately 8 x 16 cm each. In a 2x2 design, corals from each of the two reefs were transplanted either back to their reef of origin or to the other reef (i.e. fragments from offshore transplanted to inshore site) (n = 40)(Fig 1). Each small fragment was sampled in winter and summer for two years (i.e. February and August of 2012 and 2013) to capture gene expression during winter and summer conditions. Sub-samples from six of the small coral fragments from each treatment within the reciprocal transplant experiment were randomly chosen for qRT-PCR analysis for a total of n = 96 sub-samples. At each sub-sampling time, approximately 1 cm^2^ sub-samples were taken with hammer and chisel and flash frozen in liquid nitrogen within 15 minutes of initial collection and stored at -80°C.

At the end of the field experiment, *P*. *astreoides* fragments originating and replaced at Birthday (n = 6) and Acer24 (n = 6) reef were brought back to Mote Marine Tropical Laboratory (MMTL). Upon arrival, all 12 colony fragments were sectioned into nine, approximately 2x2 cm fragments each, for a total of 108 samples (54 per site) and allowed to recover for 72 hours [40] in a shaded flow through water table prior to use in the laboratory experiment discussed herein. The nine pieces resulting from each of the 12 fragments were used to accommodate a laboratory design consisting of three fragments for controls and three fragments for each of two treatment levels described below.

### Temperature and LPS experiments

The laboratory experiment consisted of a one-way design with three experimental levels: a control temperature (28°C, “Control”), a temperature known to cause bleaching (32°C, “Heat”) and a treatment in which both the bleaching temperature and *S*. *marsescens* lipopolysaccharide were applied (LPS: 32°C, “Heat+LPS”) (Sigma Aldrich, St. Louis MO, USA); the latter application was induced to mimic the immune systems recognition of bacteria *via* polysaccharide. Purified LPS is a common reagent used to elicit immune responses in human and animal studies [39]. We chose to use purified LPS instead of *S*. *marsescens* colonies to minimize potential cross contamination of *S*. *marsescens* into the surrounding environment, as MMTL’s flow-through system is connected directly to an adjacent public waterway. Additionally, LPS was not added to flow-through sections of the experimental design, see below. Control fragments were maintained in experimental tanks at ambient temperature (28°C) reflective of temperatures experienced in water tables, without exposure to LPS. Each treatment was replicated in two tanks, as outlined below, for six independent units of analysis.

Experiments were conducted by placing coral fragments in custom made acrylic boxes (2.5 x 7.5 x 7.5 cm) set within 38-liter fish tanks. The acrylic boxes included three distinct subdivisions each of which housed a single coral fragment. Six 38-liter tanks containing five acrylic boxes each were used in the experiment for n = 90 experimental units. The experimental designs for the six tanks included two controls and two tanks for each of the two treatments. The six 38-liter tanks were filled with artificial sterile seawater (ASSW, Instant Ocean, Blacksburg VA, USA) and placed in flow through water tables adjacent to one another. Water tables were 680 liters, fiberglass troughs with seawater pumped through *via* the adjacent waterway to aid in temperature maintenance.

Coral fragments were rinsed in ASSW prior to placement in acrylic box subsections. ASSW held within the tanks was circulated *via* pumps inside the tanks but external to acrylic boxes. Heaters placed external to the acrylic box maintained temperatures characteristic of bleaching (32°C±0.5) and were monitored with HOBO tags (Onset Computer Corporation, Bourne MA, USA). For the disease response treatment, LPS from *S*. *marsescens* (1 mL, 5 μg/mL) [41] was added using disposable plastic pipettes. Additionally, 1 mL of ASSW was added to the heat treatment and control tanks for consistency.

Coral fragments were exposed to treatments for eight hours. Within that period of time, individual subdivisions were aerated each hour *via* air infusion using disposable pipettes. Water was aerated (C. Page MMTL, per. obs.) taking care not to disturb the fragments. At the end of each experiment, coral fragments were removed from their individual tanks using sterile tongs, wrapped in combusted foil and placed in freezer bags. Coral fragments were then immediately flash frozen in liquid nitrogen and stored at -80°C until processing.

### RNA extraction and cDNA preparation

All sub-samples of *P*. *astreoides* were stored at -80°C and crushed to a fine powder in liquid nitrogen using a pre-chilled mortar and pestle. Between samples, mortar and pestles were cleaned with liquid detergent and RNase AWAY (Sigma-Aldrich, St. Louis MO, USA) and rinsed in ultrapure water.

TRI Reagent (Sigma-Aldrich, St. Louis MO, USA) was used to extract RNA from 0.1 g coral powder following a modified manufacturer’s protocol. Extracted RNA was quantified and two-50 μL samples were stored at -80°C. Prior to reverse transcription, samples of RNA were diluted to 5 ng/μL and examined on a bioanalyzer to check for RNA integrity and re-extracted if necessary. Once proper integrity was obtained, 2.5 ng of RNA was treated with DNase I (Life Technologies, San Antonio TX, USA) and reverse transcribed into cDNA following manufacturer instructions using the SuperScript III First-Strand Synthesis SuperMix for qRT-PCR (Life Technologies, San Antonio TX, USA).

### qRT-PCR

Fully transcribed cDNA was diluted 1:16 for qRT-PCR. Reaction volumes for qRT-PCR were 10 μL and as follows: 5 μL Power SYBR Green PCR Master Mix, 3.5 μL H_2_O, 1μL cDNA, 0.5μL primer (0.05mM final primer concentration). Cycling conditions for all reactions were: 50°C-2 minutes (1x), 95°C-10 minutes (1x), 95°C-15 seconds and 60°C-1 minute (40x). Primers were designed using Primer-BLAST (NCBI). Primer specificity parameters ignoring targets with six or more mismatches was ignored to insure primer specificity. More detailed primer information for all genes can be found in Tables 1 and 2.

**Table 1:** Genes targeted for qRT PCR.

Gene	Abbreviation	Function	References	GenBank Acc.
Cytochrome Oxidase Subunit 1	Cox	Housekeeping Gene	Rosic et al. 2011	EH037972
Proliferating Cell Nuclear Antigen	PCNA	Housekeeping Gene	McGinley et al. 2012	HE999712.2
Non-Specific Lipid-Transfer Protein	SCP2	Lipid Transfer	DeSalvo et al. 2008	DR987812
Glyceraldehyde-3-Phosphate Dehydrogenase	G3PD	Metabolism	Rosic et al. 2011	AY314974.1
Peridinin Chlorophyll a-Binding Protein 1	PCP	Photosynthesis	Jiang et al. 2012	JC395030.1
Photosystem I Reaction Center Subunit IV	PSAE	Photosystem II	Schliep et al. 2015	Zoma_C_c25681

Gene name, abbreviation, function, references, GenBank accession numbers for primers used in qRT-PCR.

**Table 2:** Primer information for qRT-PCR.

	Forward Primer		Reverse Primer				
Gene	Sequence (5'-3')	Melting Temp (°C)	Sequence (5'-3')	Melting Temp (°C)		Amplicon Length (bp)	Primer Efficiency
Cox	GGTCATTTCCATAATAATTTCTGGTGTT	60.0	CAAGACCTCCAAGAAGAGAAATAGATG		59.0	101	1.95
PCNA	GGACGTGGTGAACTTCCAGT	60.0	CCACCTTGTAGTGCTGCTCA		60.0	116	1.91
SCP2	CCAGAAGCTCCAGTCCATTC	59.8	GAGTTGCCCGGATTACACAT		59.8	110	1.76
G3PD	ACGACAAGGCCAACCATAAC	59.9	AGGAGTGGATGGTGGTCATC		59.9	129	1.80
PCP	GACTGGACCTCCGACGTTTA	60.1	GTCCATTGAAGCACCCATCT		59.9	99	1.98
PSAE	ACCTGGTGAGGTTTGAATGG	59.8	CGAAGCCAAGTACAGGAAGG		59.9	122	1.94

Abbreviations, primer sequences, melting temperature and amplicon length for gene primers used for qRT-PCR are listed in the table above.

In this study, six zooxanthellae-specific genes were analyzed across all sub-samples, and include one housekeeping gene and five experimental genes (Table 1). The housekeeping genes, cytochrome oxidase subunit 1 (*Cox*) and proliferating cell nuclear antigen (*PCNA*) have previously been identified as viable housekeeping genes in *Symbiodinium* spp. [42,43]. Technical replicates were run in duplicate and negative controls were run to confirm no genomic DNA contamination for a total of n = 2,448 reactions. All analyses were completed at the Research Technology Support Facility at Michigan State University on an Applied Biosystems Prism 7900HT (Thermo Scientific, New York, NY USA).

### Data analysis

All analyses were performed in R (v3.1.3) using the specialized package *MCMC*.*qpcr* [44]. In this analysis, raw qRT-PCR data (i.e. Ct values) is represented as molecule counts and described under a Poisson-lognormal error using generalized linear mixed models. There are numerous benefits to this approach. It is fully flexible for all levels of random and fixed effects, enables evaluation of unlimited interactions, increases power *via* simultaneous analysis of all genes in one model, accounts for low amplification targets by using molecule counts, and eliminates the need for control genes [44]. For this study, however, control genes were used to balance conservatism and the risk of bias, as suggested by Matz *et al*.[44].

For analysis of field samples, a two-way model was fit using “treatment” (i.e. transplant versus non-transplant) and “time” (i.e. winter and summer) as fixed factors, as well as their interaction, “treatment:time”. This resulted in a model describing the individual effect of each factor, as well as the effect of their interaction (i.e. the treatment dependent effect of sampling time). Two models were run in order to report all relevant comparisons. The first model used fragments originating from, and replaced at, Acer24 reef as a baseline comparison, meaning all other fragment conditions were compared to fragments originating from and replaced at, Acer24 reef. The second model used fragments originating and replaced at Birthday reef for baseline comparison. Three Markov Chain Monte Carlo chains were run for 25,000 iterations with the first 4,000 discarded as burn-in. Additionally, the model was “informed” using qRT-PCR data generated here from one previously established control gene (*Cox*, Table 1, [42]) and used original coral fragment and individual samples as random variables to account for potential sources of variation and the repeated sampling design [44]. Diagnostic plots using the function diagnostic.mcmc() were analyzed to confirm linear modeling as an appropriate application for this data set.

For analysis of laboratory samples, a two-way model was fit using “reef” (i.e. Acer24 or Birthday reef) and “treatment” (i.e. control, heat or heat+LPS) as fixed factors, as well as their interaction term, “reef:treatment”. This resulted in a model describing the individual effects of each factor, in addition to the interaction effect (i.e. the site dependent effect of treatment). Three Markov Chain Monte Carlo chains were run for 25,000 iterations with the first 4,000 discarded as burn-in. The model was “informed” using qRT-PCR data generated here from one previously established housekeeping gene (*PCNA*, [43]) and used original coral fragment, individual samples and tank assignment as random variables to account for potential sources of variation[44]. Diagnostic plots using the function diagnostic.mcmc() were analyzed to confirm linear modeling as an appropriate application for this data set.

## Results

### Reciprocal transplant

**Fig 2:**
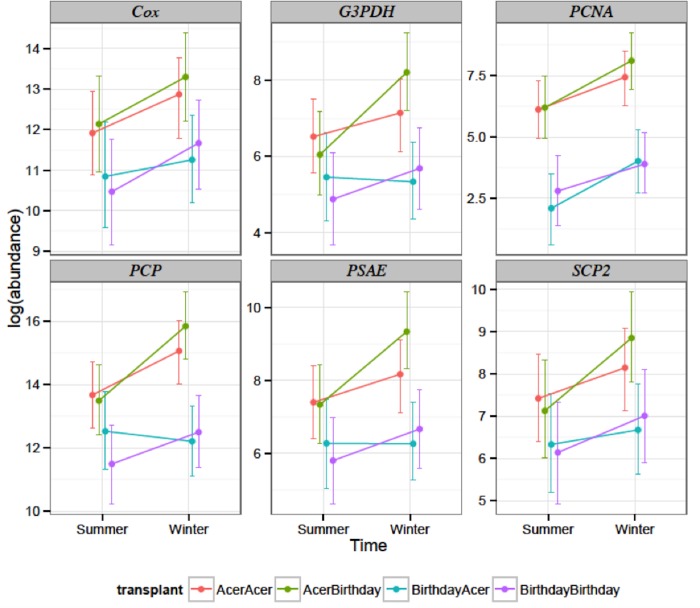
By-gene plot of transcript abundance for zooxanthellae genes obtained from sub-samples of *Porites* astreoides taken from Birthday reef and Acer24 reef during winter and summer sampling efforts. “AcerAcer” represents non-transplanted sub-samples that originated at Acer24 reef, whereas “AcerBirthday” represents transplanted sub-samples that were collected at Acer24 reef and transplanted to Birthday reef. “BirthdayBirthday” represents non-transplanted sub-samples that originated at Birthday reef, whereas “BirthdayAcer” represents transplanted sub-samples that were collected at Birthday reef and transplanted to Acer24 reef. Sub-samples collected in summer months are represented in orange, while sub-samples collected in winter months are represented in blue. Whiskers denote 95% credible intervals.

There were no significant effects in gene expression between winter and summer observed, however, significant differences in zooxanthellae gene expression were found between sub-samples from Acer24 and Birthday reefs (p<0.05) (Table 3, Table 4, S1 File). Overall, gene expression from zooxanthellae was higher in algae from Acer24 reef than those present at Birthday reef (Fig 2). Gene expression patterns observed in zooxanthellae from the original site were retained in the symbiont throughout the transplant period for both collection reefs. For example, gene expression patterns of *Symbiodinium* spp. from Acer24 reef that were transplanted to Birthday reef were not significantly different from patterns of zooxanthellae that were from, and held, at Acer24 reef (Fig 2).

**Table 3:** Summary of statistical output of zooxanthellae gene expression using AcerAcer as a reference factor.

Sample	Gene	Post.mean	l-95% CI	u-95% CI	Eff.samp	pMCMC	
AcerBirthday	G3PD	0.75786	-0.32954	1.81690	1100.1	0.182	
AcerBirthday	PCP	0.57277	-0.65011	1.58568	1000.0	0.334	
AcerBirthday	PCNA	0.45110	-0.85448	1.74646	1000.0	0.472	
AcerBirthday	PSAE	0.82805	-0.24776	1.92126	1095.1	0.146	
AcerBirthday	SCP2	0.51517	-0.62875	1.57658	1140.2	0.368	
AcerBirthday	Cox	0.29714	-0.81822	1.34287	1000.0	0.598	
BirthdayAcer	G3PD	-1.27444	-2.32264	-0.17412	1000.0	0.024	*
BirthdayAcer	PCP	-1.99936	-3.00025	-0.71156	1000.0	<0.001	*
BirthdayAcer	PCNA	-2.41345	-3.72389	-1.14470	1000.0	<0.001	*
BirthdayAcer	PSAE	-1.33718	-2.43305	-0.23223	1000.0	0.018	*
BirthdayAcer	SCP2	-1.04736	-2.08630	0.05666	1000.0	0.072	
BirthdayAcer	Cox	-1.15290	-2.32132	-0.11005	1000.0	0.056	
BirthdayBirthday	G3PD	-1.03375	-2.11422	-0.03793	1024.5	0.052	
BirthdayBirthday	PCP	-1.79766	-2.94654	-0.74262	1122.8	0.002	*
BirthdayBirthday	PCNA	-2.49322	-3.63201	-1.13299	1000.0	<0.001	*
BirthdayBirthday	PSAE	-1.05099	-2.07456	-0.01014	1063.0	0.044	*
BirthdayBirthday	SCP2	-0.80628	-1.87348	0.26407	1046.7	0.156	
BirthdayBirthday	Cox	-0.86286	-1.96599	0.21116	1034.0	0.140	
Summer	G3PD	-0.42471	-1.44246	0.57653	1000.0	0.400	
Summer	PCP	-0.96875	-2.08771	0.07127	1000.0	0.076	
Summer	PCNA	-0.90811	-2.04225	0.42268	1000.0	0.136	
Summer	PSAE	-0.53284	-1.49807	0.53535	1000.0	0.298	
Summer	SCP2	-0.50555	-1.67745	0.41615	1000.0	0.336	
Summer	Cox	-0.66882	-1.56163	0.42765	1000.0	0.176	
AcerBirthday:Summer	G3PD	-1.10678	-2.53604	0.23038	1000.0	0.146	
AcerBirthday:Summer	PCP	-0.71755	-2.36609	0.71800	1000.0	0.366	
AcerBirthday:Summer	PCNA	-0.42148	-2.29178	1.34468	1102.2	0.660	
AcerBirthday:Summer	PSAE	-0.88605	-2.41830	0.49638	1000.0	0.258	
AcerBirthday:Summer	SCP2	-0.73566	-2.13112	0.78400	1000.0	0.346	
AcerBirthday:Summer	Cox	-0.15287	-1.43671	1.36536	1000.0	0.860	
BirthdayAcer:Summer	G3PD	0.48370	-1.05469	1.93171	1000.0	0.528	
BirthdayAcer:Summer	PCP	1.17241	-0.36614	2.84003	1000.0	0.162	
BirthdayAcer:Summer	PCNA	-0.44314	-2.25117	1.56216	1000.0	0.648	
BirthdayAcer:Summer	PSAE	0.51274	-1.06876	2.00907	1043.4	0.506	
BirthdayAcer:Summer	SCP2	0.24851	-1.14452	1.85562	1000.0	0.748	
BirthdayAcer:Summer	Cox	0.35344	-1.07449	1.79065	1121.9	0.672	
BirthdayBirthday:Summer	G3PD	-0.11873	-1.52551	1.26020	1000.0	0.860	
BirthdayBirthday:Summer	PCP	0.28191	-1.21268	1.80808	1000.0	0.704	
BirthdayBirthday:Summer	PCNA	0.17796	-1.53111	2.15363	1000.0	0.822	
BirthdayBirthday:Summer	PSAE	-0.06285	-1.50997	1.28971	1000.0	0.940	
BirthdayBirthday:Summer	SCP2	-0.08662	-1.60147	1.34879	1000.0	0.900	
BirthdayBirthday:Summer	Cox	-0.16014	-1.51131	1.16904	1000.0	0.818	

Output for two-way model using “time”, “transplant” and their interaction as fixed factors under the MCMC.qpr package for zooxanthellae gene expression from subsamples collected at Birthday reef and Acer24 reef. Post means are reported as well as lower and upper credible intervals, effective sample size and p-values. “AcerAcer” represents non-transplanted sub-samples that originated at Acer24 reef, whereas “AcerBirthday” represents transplanted sub-samples that were collected at Acer24 reef and transplanted to Birthday reef. “BirthdayBirthday” represents non-transplanted sub-samples that originated at Birthday reef, whereas “BirthdayAcer” represents transplanted sub-samples that were collected at Birthday reef and transplanted to Acer24 reef. Baseline comparison, or reference factor, included non-transplanted sub-samples from Acer24 reef (i.e. AcerAcer) at the winter sampling time. The model was “informed” using *Cox* as a housekeeping gene and run with 25,000 iterations, with the first 4,000 discarded as burn-in. Asterisk denotes a p-value <0.05.

**Table 4:** Summary of statistical output of zooxanthellae gene expression using BirthdayBirthday as a reference factor.

Sample	Gene	Post.mean	l-95% CI	u-95% CI	Eff.samp	pMCMC	
AcerAcer	G3PD	1.21755	0.17023	2.20626	1000.0	0.022	*
AcerAcer	PCP	1.99231	0.96816	3.19196	1000.0	<0.001	*
AcerAcer	PCNA	2.72957	1.49594	3.84646	1000.0	<0.001	*
AcerAcer	PSAE	1.25442	0.20186	2.25162	1000.0	0.022	*
AcerAcer	SCP2	1.00775	-0.02516	2.11803	1000.0	0.080	
AcerAcer	Cox	1.09524	0.05914	2.19882	1000.0	0.056	
AcerBirthday	G3PD	1.64635	0.54220	2.66718	1000.0	<0.001	*
AcerBirthday	PCP	2.22900	1.03429	3.28904	1000.0	<0.001	*
AcerBirthday	PCNA	2.83415	1.54539	4.01819	1000.0	<0.001	*
AcerBirthday	PSAE	1.74730	0.65014	2.81450	1000.0	0.002	*
AcerBirthday	SCP2	1.18808	0.16333	2.30082	1000.0	0.028	*
AcerBirthday	Cox	0.98921	-0.13981	2.08998	909.6	0.094	
BirthdayAcer	G3PD	-0.31557	-1.38858	0.74282	1092.6	0.580	
BirthdayAcer	PCP	-0.27211	-1.36499	0.85086	1000.0	0.650	
BirthdayAcer	PCNA	0.04449	-1.31269	1.16807	1136.6	0.934	
BirthdayAcer	PSAE	-0.35742	-1.51305	0.65777	943.5	0.518	
BirthdayAcer	SCP2	-0.30484	-1.42683	0.71932	1113.7	0.600	
BirthdayAcer	Cox	-0.41275	-1.52753	0.68653	1098.6	0.444	
Summer	G3PD	-0.42119	-1.38320	0.59371	1000.0	0.410	
Summer	PCP	-0.56929	-1.60197	0.51766	1000.0	0.300	
Summer	PCNA	-0.50935	-1.64950	0.90378	1110.9	0.414	
Summer	PSAE	-0.47024	-1.42073	0.61740	1107.8	0.370	
Summer	SCP2	-0.46287	-1.44652	0.61586	1000.0	0.398	
Summer	Cox	-0.69225	-1.64933	0.33131	1000.0	0.166	
AcerAcer:Summer	G3PD	-0.27623	-1.60932	1.10719	933.0	0.716	
AcerAcer:Summer	PCP	-0.67608	-2.14837	0.73583	888.2	0.388	
AcerAcer:Summer	PCNA	-0.68121	-2.33178	0.95846	1000.0	0.446	
AcerAcer:Summer	PSAE	-0.33763	-1.68846	1.13446	921.6	0.654	
AcerAcer:Summer	SCP2	-0.32461	-1.65484	1.17265	887.0	0.652	
AcerAcer:Summer	Cox	-0.31844	-1.64613	1.04231	959.7	0.658	
AcerBirthday:Summer	G3PD	-0.98030	-2.38653	0.45556	1000.0	0.182	
AcerBirthday:Summer	PCP	-0.97558	-2.51798	0.46522	1000.0	0.208	
AcerBirthday:Summer	PCNA	-0.60709	-2.36205	1.04494	1000.0	0.502	
AcerBirthday:Summer	PSAE	-0.81838	-2.28514	0.60443	1000.0	0.272	
AcerBirthday:Summer	SCP2	-0.65107	-2.10597	0.78232	1000.0	0.390	
AcerBirthday:Summer	Cox	0.01370	-1.42340	1.33326	1000.0	0.986	
BirthdayAcer:Summer	G3PD	0.52894	-0.79296	1.88105	1150.7	0.456	
BirthdayAcer:Summer	PCP	0.82061	-0.72111	2.21444	1125.8	0.278	
BirthdayAcer:Summer	PCNA	-0.75939	-2.51604	0.91120	1146.2	0.406	
BirthdayAcer:Summer	PSAE	0.51246	-0.88945	1.90230	1126.0	0.480	
BirthdayAcer:Summer	SCP2	0.25853	-1.00056	1.84046	1000.0	0.738	
BirthdayAcer:Summer	Cox	0.46346	-0.91224	1.83777	1125.6	0.506	

Output for two-way model using “time” and “transplant” as fixed factors under the MCMC.qpr package for zooxanthellae gene expression from sub-samples collected at Birthday reef and Acer24 reef. Post means are reported as well as lower and upper credible intervals, effective sample size and p-values. “AcerAcer” represents non-transplanted sub-samples that originated at Acer24 reef, whereas “AcerBirthday” represents transplanted sub-samples that were collected at Acer24 reef and transplanted to Birthday reef. “BirthdayBirthday” represents non-transplanted sub-samples that originated at Birthday reef, whereas “BirthdayAcer” represents transplanted sub-samples that were collected at Birthday reef and transplanted to Acer24 reef. Baseline comparison, or reference factor, included non-transplanted sub-samples from Birthday reef (i.e. BirthdayBirthday) at the winter sampling time. The model was “informed” using *Cox* as a housekeeping gene and run with 25,000 iterations, with the first 4,000 discarded as burn-in. Asterisk denotes a p-value <0.05.

Several genes from zooxanthellae harbored in *P*. *astreoides* sub-samples originating from Birthday reef exhibited a significant down regulation relative to symbionts originating from Acer24 reef. These included *PCP* (p = 0.002), *PCNA* (p = <0.001) and *psaE* (p = 0.044) and *G3PDH* (p = 0.052) (Fig 2, Table 3). Relative to zooxanthellae from sub-samples originating at Acer24 reef, *Symbiodinium* spp. originating from Birthday reef and transplanted to Acer24 reef exhibited significant down-regulation of the genes *PCP* (p<0.001), *PCNA* (p<0.001), *psaE* (p = 0.018), and *G3PDH* (p = 0.024)(Fig 2, Table 3). Relative to zooxanthellae originating at Birthday reef, *Symbiodinium* spp. originating at Acer24 reef and transplanted to Birthday reef exhibited significant up-regulation of genes *PCP* (p<0.001), *PCNA* (p<0.001), *psaE* (p = 0.002), *G3PDH* (p<0.001) and *SCP2* (p = 0.028)(Fig 2, Table 3).

In contrast to above, no significant effect of site was found when comparing zooxanthellae gene expression from sub-samples originating at Acer24 reef and zooxanthellae originating from Acer24 reef sub-samples transplanted to Birthday reef (p>0.05, Table 3). Furthermore, gene expression did not vary significantly between zooxanthellae originating from Birthday reef sub-samples and zooxanthellae originating from Birthday reef sub-samples that were transplanted to Acer24 reef (Table 3).

For all of the zooxanthellae genes tested throughout all experimental conditions, expression was higher in winter, compared to summer, but the effect of season was not significant (p>0.07, Fig 2, Tables 3 & 4). In zooxanthellae from Birthday reef sub-samples transplanted to Acer24 reef, *G3PDH* and *PCP* experienced slight decreased expression in winter compared to summer, while *psaE* experienced no change in expression. Finally, the interaction between time and transplant was not significant (p>0.1, Tables 3 & 4).

### Temperature and LPS experiments

No significant effect of site or treatment was found on the expression of *Cox*, *G3PDH*, *SCP2*, *PCP* or *psaE* between Acer24 and Birthday reef zooxanthellae associated with the coral fragments (p>0.05, Table 5, S2 File). For example, gene expression of *PCP* was not different between Acer24 and Birthday reef fragments or between control and experimental treatments from the same site. The interaction of site and treatment also did not have a significant effect on zooxanthellae expression (p>0.05, Table 5). For instance, the gene expression of *PCP* in heat-stressed *Symbiodinium* spp. from Acer24 reef was not different from the expression of *PCP* heat-stressed zooxanthellae from Birthday reef. Pair-wise comparisons showed no significant differences in gene expression among treatments and controls. The expression of *PCP* in zooxanthellae harbored in control fragments from Birthday reef was not significantly different from the expression of *PCP* in zooxanthellae harbored in fragments from Birthday reef exposed to heat or heat+LPS treatments (p>0.05).

**Table 5:** Summary of statistical output of zooxanthellae gene expression in temperature LPS experiments.

Treatment	Gene	Post.mean	l-95% CI	u-95% CI	Eff.samp	pMCMC
Reef	Cox	2.187638	-0.139690	4.479140	1000.0	0.068
Reef	G3PDH	0.504446	-0.644153	1.417098	1000.0	0.334
Reef	PCP	0.245573	-0.812485	1.259310	1000.0	0.636
Reef	PSAE	0.038782	-0.910168	1.093089	1000.0	0.914
Reef	SCP2	1.174183	-0.081910	2.613642	1000.0	0.102
Reef	PCNA	0.353228	-0.635999	1.392324	1000.0	0.538
Heat	Cox	0.631882	-1.126961	2.276879	1000.0	0.452
Heat	G3PDH	0.370865	-0.632919	1.328137	1000.0	0.466
Heat	PCP	0.410617	-0.656092	1.311776	1000.0	0.430
Heat	PSAE	0.418968	-0.651622	1.390965	1000.0	0.414
Heat	SCP2	0.603772	-0.547622	1.572087	1000.0	0.300
Heat	PCNA	0.024704	-0.753097	0.877509	1000.0	0.980
LPS	Cox	0.007293	-1.872295	1.749692	898.7	0.986
LPS	G3PDH	0.345562	-0.654212	1.385999	1000.0	0.520
LPS	PCP	0.613552	-0.434824	1.643339	798.5	0.262
LPS	PSAE	0.556251	-0.492400	1.607295	799.0	0.314
LPS	SCP2	0.223214	-0.903428	1.304645	1000.0	0.686
LPS	PCNA	-0.001164	-0.869460	0.914153	1000.0	0.982
Reef+Heat	Cox	-1.832442	-4.065119	0.415856	872.3	0.094
Reef+Heat	G3PDH	-0.088287	-1.412416	1.204037	1000.0	0.904
Reef+Heat	PCP	-0.061582	-1.457569	1.138234	709.4	0.916
Reef+Heat	PSAE	0.009594	-1.246905	1.411503	732.4	0.994
Reef+Heat	SCP2	-0.163576	-1.403211	1.327639	1000.0	0.816
Reef+Heat	PCNA	-0.145029	-1.095284	0.821031	867.0	0.768
Reef+LPS	Cox	-0.438337	-2.817670	1.779693	1000.0	0.712
Reef+LPS	G3PDH	-0.110682	-1.423377	1.182371	1000.0	0.848
Reef+LPS	PCP	-0.414284	-1.808073	0.857795	1000.0	0.526
Reef+LPS	PSAE	-0.086955	-1.474356	1.181806	1000.0	0.886
Reef+LPS	SCP2	0.069792	-1.298356	1.489236	1000.0	0.918
Reef+LPS	PCNA	-0.126965	-0.963841	0.885971	1121.8	0.770

Two-way model for an experiment testing the effects of reef, heat and heat+LPS on the gene expression of *Cox*, *G3PDH*, *PCP*, *psaE*, *SCP2* and *PCNA* under the MCMC.qpr package. Post means are reported as well as lower and upper credible intervals, effective sample size and p-values. The model was “informed” using *PCNA* as a housekeeping gene and run with 25,000 iterations, with the first 4,000 discarded as burn-in. Coral colony individual was used as a random factor. Reef fragments were from Acer24 or Birthday reef and exposed to 28°C. Heat treatments were fragments from both reefs exposed to 32°C, and heat+LPS treatments were fragments from both reefs exposed to 32°C+LPS from *Serratia marcescens*. Asterisk denotes a p-value <0.05.

**Fig 3:**
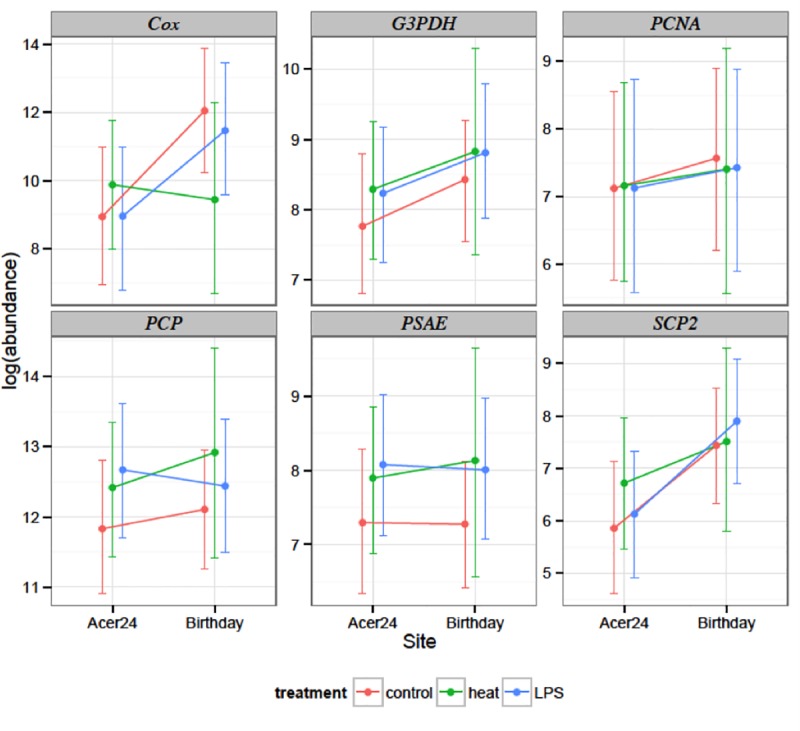
By-gene plot of normalized log_2_-transformed expression values (±SEM) of experimental genes from all samples. Orange lines represent fragments exposed to control treatments (28°C), green lines represent fragments exposed to heat treatments (32°C) and blue lines represent fragments exposed to heat+LPS treatments (32°C+LPS from *Serratia marcescens*). Whiskers denote 95% credible intervals.

Although not significant, the cumulative effect of heat stress on the expression of each of these genes generally resulted in an increase in gene expression relative to controls. For some genes (e.g. *Cox*), an increase in temperature resulted in a decrease in expression (Fig 3). The only other time a decrease in gene expression was observed was when *PCP* and *PSAE* were exposed to the heat+LPS treatment. In addition, depending upon treatment, the stress resulted in a 2-fold increase in expression relative to the control (e.g. *Cox*, *SCP2*; Fig 3)

## Discussion

Gene expression analysis is a powerful tool increasingly utilized to determine responses of closely related coral and symbiotic zooxanthellae populations to stress [35,36,43,45]. Coral host populations of *P*. *astreoides* exhibit differential gene expression that has been linked to bleaching tolerance [14]. To understand the role of zooxanthellae from *P*. *astreoides* in bleaching susceptibility at Acer24 and Birthday reefs characterized by Haslun *et al*.[16], we studied gene expression in *Symbiodinium* spp., from these reefs, as well as a reciprocal transplant. Results demonstrating increased gene expression in zooxanthellae from the offshore reef, which experiences higher bleaching, and no change in gene expression patterns in reciprocal transplants, allowed us to make inferences regarding the roles of zooxanthellae and acclimatization in bleaching susceptibility.

Differences in zooxanthellae gene expression between *P*. *astreoides* sub-samples from inshore and offshore sites are indicative of functional variability within zooxanthellae. Elevated gene responses and higher rates of bleaching seen at Acer24 reef relative to inshore may be a consequence of higher irradiance [16,46–48]. Owing to the elevated coral-host stress response at Acer24 reef, we interpreted patterns of zooxanthellae gene expression exhibited in Acer24 reef sub-samples to reflect bleaching susceptibility at the cellular level. With the exception of *SCP2* in zooxanthellae from sub-samples originating and retained at Acer24 reef, four symbiont genes (*PCNA*, *G3PDH*, *PCP* and *psaE*) displayed significantly higher expression in all offshore Acer24 reef sub-samples compared to inshore Birthday reef sub-samples (Tables 3 & 4). These zooxanthellae genes are associated with metabolic processes that increase with stress, such as DNA synthesis, lipid transfer, metabolism and photosynthesis [37,49,50]. Consequently, increased zooxanthellae gene expression observed at Acer24 reef may be a reflection of differential bleaching susceptibility experienced by the symbiont.

Although normally involved in glycolysis, *G3PDH* expression has been associated with apoptosis activation [51]. Apoptosis, also referred to as programmed cell death, occurs during the beginning stages of host coral bleaching [52,53]. The possibility of increased zooxanthellae *G3PDH* expression signaling symbiont apoptosis and subsequent bleaching in coral originating at Acer24 reef is consistent with significantly higher levels of bleaching among host coral fragments originating from Acer24 reef relative to those originating from Birthday reef [16].

Increased expression of *PCNA* and *SCP2* in zooxanthellae from Acer 24 reef suggests higher rates of DNA synthesis and lipid transfer/synthesis, processes that occur during cell replication and division. Our observation of increased expression of *PCNA* and *SCP2* suggests that rates of zooxanthellae replication and division increases with elevated stress [54]. During a bleaching event in which stress results in host bleaching, it is plausible that the demands placed upon the symbiont *via* the host results in increased zooxanthellae lipid metabolism and cellular division [55]. As a consequence, the symbiont increases lipid use not only as a mechanism to increase its own metabolism, but also to increase lipid transfer to the host [56].

Rates of photosynthesis increase in response to moderate stress in cultured *Symbiodinium* spp. [57]. The increased expression of *PCP* in Acer24 reef samples is consistent with this observation, as *PCP* is related to light harvesting. In addition to increased *PCP*, *PsaE*, a protein involved in the stabilization of interactions within the photosystem 1 complex (PSI), reflects elevated expression in zooxanthellae from Acer24 sub-samples relative to those at Birthday reef. *PsaE* expression increases in the cyanobacterium *Synechocystis* spp. during light stress [58]. The increases in *psaE* expression counteracts the effects of reactive oxygen species (ROS), whose presence breaks down photosystem II machinery [59]. ROS production is a common response of *Symbiodinium* spp. to bleaching conditions [60]. Increases in the expression of stabilizing protein *psaE* in Acer24 reef samples may represent an initial attempt to counteract degradation of photosynthetic machinery repair as reported in McGinley *et al*. [43] for *Symbiodinium* spp. A13. Increase in the expression of *psaE* also demonstrates the higher stress environment offshore.

The lack of an effect of transplantation and the retention of zooxanthellae gene expression patterns from reef of origin, demonstrates that environmental change does not influence the expression patterns of zooxanthellae genes investigated in this study. If acclimatization were an important means of counteracting bleaching susceptibility, zooxanthellae gene expression patterns would have likely changed in the transplant experiment. Instead, gene expression may be genetically determined by adaptation, as was also proposed for the coral host [15]. The lack of acclimatization demonstrated in gene expression parallels previous research demonstrating a lack of change in zooxanthellae subclade type populations associated with *P*. *astreoides* from Acer24 and Birthday reefs in response to reciprocal transplant [17]. Differences in zooxanthellae subclade populations seen in Hauff *et al*. [17] reflect variation in genetic material for natural selection to act upon, and may have resulted in locally adapted populations of zooxanthellae at Acer24 and Birthday reefs. Although demonstrating a lack of acclimatization to environmental change, gene expression results suggest that zooxanthellae associated with *P*. *astreoides* in the Florida Keys are functionally variable and the lower expression of stress response genes relative to the offshore reef likely reflects better adaptation to bleaching susceptibility inshore.

While zooxanthellae from *P*. *astreoides* demonstrated functional variation in the field, the continued success of coral reefs will likely be determined by the ability of the coral-zooxanthellae association to survive exposure to chronic moderate stress and acute extreme bleaching events [3,13]. While chronic moderate stress is commonly experienced by zooxanthellae on reefs in the Florida Keys [16,23], acute stress is a more transient phenomenon poorly described in the literature. Most studies report a response of the coral host and imply that such effects also impact the metabolism of the symbionts [15,61]. To evaluate the influence of acute stress on zooxanthellae gene response we exposed the same coral from Acer24 and Birthday reefs to a high temperature (32°C) stress, and the same high temperature combined with LPS from *S*. *marsescens*; a treatment that represents a primary stress coupled with a secondary stress. Although data was not statistically significant (Table 5), cumulative trends demonstrate increased expression of five of the six zooxanthellae genes relative to the experimental control in response to heat stress, a trend also found in the literature [62]. During acute temperature exposure, symbiont cells show a “knee-jerk” reaction in which cumulative gene expression increases one or two-fold, increasing photosynthetic capacity, DNA synthesis and lipid metabolism (Fig 3)[62,63].

We speculate the lack of a symbiont gene response is likely due to the coral host influencing zooxanthellae gene expression by providing protection to zooxanthellae. This possibility is suggested by the observation that *in hospite* zooxanthellae experience less photosynthetic degradation than cultured zooxanthellae when exposed to short-term bleaching conditions [64]. Because the viability of zooxanthellae metabolism is not independent of the coral host, understanding the response of the coral host to heat and disease conditions is relevant to the current study. Using the same samples as this study, Haslun *et al*. (In Review) evaluated the genetic response of the coral host. Coral host genes associated with LPS recognition increased under acute heat and LPS stress. This suggests that coral, rather than zooxanthellae, are the first responders to acute stress. Additionally, as the current study modeled the response of zooxanthellae to LPS exposure, and not a bacterial infection, it is possible the 8-hour sampling period was too long to observe a proper response. The response of the innate immune system to LPS molecules is very rapid, and may not have been captured after eight hours [65]. Deciphering which symbiotic partner experiences the lions-share of acute stress [66] will be key in determining the range at which coral holobionts can resist acute extreme episodes of stress.

Short-term perturbations such as acute stress are difficult to capture in the field. As a counterpart to field studies, laboratory studies are important as they allow for manipulation and the capture of acute stress perturbations. This study demonstrated an absence of statistically significant functional variability to acute stress, even though functional variation in zooxanthellae populations from *P*. *astreoides* as a consequence of chronic stress was established under similar or less stressful conditions in the field. The role of the host as a protective mechanism may explain the apparent lack of variability reported. Alternatively, the short time period may have also skewed the results, indicating longer sampling intervals are required to induce a significant stress response in zooxanthellae. The ecological implications of the field and lab findings suggest that the response of the coral holobiont to stress is dynamic. As such, the future persistence of *P*. *astreoides* is dependent on a myriad of environmental and biological parameters, as well as a complex interplay between the host and symbiont. To the authors’ knowledge, this study marks the first study of zooxanthellae gene expression in response to synergistic temperature as a primary stress coupled to LPS as a secondary stress.

## Supporting information

S1 File Gene expression data from reciprocal transplant. Ct values for corresponding genes are listed in columns 1–6. Transplant lists reef or origin and reef of transplant, respectively. Time refers to the season in which the fragment was sampled, i.e. Winter or Summer.
(CSV)

S2 File Gene expression data from temperature and LPS experiments. Ct values for corresponding genes are listed in columns 1–6. Sample refers to the coral fragment the gene corresponds to with duplicate internal replicates, while individual refers to parent coral colonies. Transplant corresponds to reef or origin (i.e. Birthday Reef or Acer Reef). Treatment refers to whether the coral experienced control,
(CSV)
